# Network Pharmacology of Yougui Pill Combined with Buzhong Yiqi Decoction for the Treatment of Sexual Dysfunction

**DOI:** 10.1155/2019/1243743

**Published:** 2019-11-11

**Authors:** Yangyun Wang, Wandong Yu, Chaoliang Shi, Wei Jiao, Junhong Li, Jianchao Ge, Yang Hong, Guowei Shi

**Affiliations:** ^1^Department of Urology, The Fifth People's Hospital of Shanghai, Fudan University, Shanghai 200240, China; ^2^Department of Pelvic Floor Center, Minhang District, Shanghai 200240, China

## Abstract

**Purpose:**

We aimed to find the possible key targets of Yougui pill and Buzhong Yiqi decoction for the treatment of sexual dysfunction.

**Materials and Methods:**

The composition of Yougui pill combined with Buzhong Yiqi decoction was obtained, and its effective components of medicine were screened using ADME; the component target proteins were predicted and screened based on the TCMSP and BATMAN databases. Target proteins were cross-validated using the CTD database. We performed gene ontology (GO) and Kyoto Encyclopedia of Genes and Genomes (KEGG) pathway analyses for target proteins using the Cytoscape plugin ClueGO + CluePedia and the R package clusterProfiler, respectively. Subsequently, protein-protein interaction (PPI) analyses were conducted using the STRING database. Finally, a pharmacological network was constructed.

**Results:**

The pharmacological network contained 89 nodes and 176 relation pairs. Among these nodes, there were 12 for herbal medicines (orange peel, licorice, *Eucommia*, *Aconite*, *Astragalus*, Chinese wolfberry, yam, dodder seed, ginseng, *Cornus officinalis*, *Rehmannia*, and *Angelica*), 9 for chemical components (18-beta-glycyrrhetinic acid, carvacrol, glycyrrhetinic acid, higenamine, nobilin, quercetin, stigmasterol, synephrine, and thymol), 62 for target proteins (e.g., NR3C1, ESR1, PTGS2, CAT, TNF, INS, and TP53), and 6 for pathways (MAPK signaling pathway, proteoglycans in cancer, dopaminergic synapse, thyroid hormone signaling pathway, cAMP signaling pathway, and neuroactive ligand-receptor interaction).

**Conclusion:**

NR3C1, ESR1, PTGS2, CAT, TNF, INS, and TP53 may be important targets for the key active elements in the decoction combining Yougui pill and Buzhong Yiqi. Furthermore, these target proteins are relevant to the treatment of sexual dysfunction, probably via pathways associated with cancer and signal transduction.

## 1. Introduction

Sexual dysfunction is difficulty experienced by a couple or an individual during any stage of a normal sexual activity [1]. It is reported that the sexual dysfunction included orgasm disorders, arousal disorders, and interest or desire disorders [2]. The two most common male sexual dysfunctions are premature ejaculation and erectile dysfunction [3]. Erectile dysfunction is defined as a persistent inability to attain or maintain an erection sufficient to permit satisfactory sexual performance [4]. Premature ejaculation is ejaculation earlier than desired and with minimal stimulation [5]. In both men and women, factors including diabetes mellitus, age, general health, cardiovascular disease, other chronic health problems, and urinary tract infections play significant roles in the development of sexual dysfunction [2]. About 5% to 20% of men suffer from moderate to severe erectile dysfunction [6], and the prevalence rate of premature ejaculation is 20% to 30% [7]. Sexual dysfunction has severe adverse effects on patient's quality of life, such as marital discord, loss of self-esteem and self-confidence, anxiety, and poor self-image [8], and therefore, effective clinical treatments for sexual dysfunction are necessary.

Yougui pill and Buzhong Yiqi are important traditional Chinese medicines (TCMs). Yougui pill decoction consists of *Rehmannia* root, dogwood fruit, yam rhizome, Chinese wolfberry, antler glue, dodder seed, *Eucommia* bark, *Angelica*, cinnamon bark, and monkshood root. Buzhong Yiqi decoction is a tonic for the middle Jiao and replenishes and raises qi [9, 10]. It is composed of *Astragalus membranaceus*, Zhigancao, *Codonopsis pilosula*, largehead atractylodes rhizome, *Angelica sinensis*, rattletop, *Radix bupleuri*, and dried tangerine or orange peel. Previous studies reveal that the combination decoction of Yougui pill and Buzhong Yiqi can be used to effectively treat sexual dysfunction [11–13]. However, the molecular mechanism has remained unclear until now.

Network pharmacology is an integrated multidisciplinary concept, and it provides a novel network mode encompassing “multiple targets, multiple effects, complex diseases” based on polypharmacology and systems biology [14]. Gui-Biao et al. have proposed network pharmacology as a new approach to the research of Chinese herbal medicine [15]. The herbal formulas of TCM are multitarget and multicomponent therapeutics; consequently, network pharmacology methods are well suited to fundamental studies of the combination rules for multiple ingredients in these prescriptions [16]. The interactions between relevant targets and active compounds are also better understood by the use of these methods [17]. In the present study, firstly, the composition of Yougui pill combined with Buzhong Yiqi decoction was obtained. The effective components of medicine were screened, and component target proteins were predicted and screened. Then, target proteins were cross-validated, and the gene ontology (GO) and Kyoto Encyclopedia of Genes and Genomes (KEGG) pathway analyses for target proteins were conducted. After that, protein-protein interaction (PPI) and pharmacological networks were constructed. We aimed to find the possible key targets of Yougui pill and Buzhong Yiqi decoction for the treatment of sexual dysfunction.

## 2. Materials and Methods

### 2.1. Composition of Traditional Chinese Medicine Preparation

The composition of the combined Yougui pill and Buzhong Yiqi decoction was as follows: *Rehmannia,* 24 g; yam, 30 g; *Cornus officinalis*, 15 g; Chinese wolfberry, 9 g; dodder, 12 g; deer horn gum, 12 g; *Eucommia*, 12 g; *Cinnamomum cassia*, 6 g; *Angelica*, 9 g; *Aconite,* 6 g; *Astragalus membranaceus,* 18 g; licorice, 9 g; ginseng, 6 g; orange peel, 6 g; *Morinda,* 15 g; *Atractylodes,* 9 g; and Xianling spleen, 15 g.

The TCMSP database (http://lsp.nwu.edu.cn/browse.php?qc=herbs) [18] was used to obtain composition information for these ingredients, including number of components, molecule name, molecular weight, fatty water partition coefficient, hydrogen bond donor acceptor number, oral bioavailability (OB), intestinal epithelial permeability, blood-brain barrier (BBB), drug-likeness (DL), and drug half-life (HL).

In addition, TCMID database (http://183.129.215.33/tcmid/search/) was used to search relevant information of that was not included in the TCMSP database.

### 2.2. Screening of Effective Components of Medicine

Screening for possible small drug molecules within the combined decoction was performed, based on absorption, distribution, metabolism, and excretion (ADME) parameters [19, 20] from the TCMSP database, where available, and from the TCMID database if not. The ADME parameters used were OB, DL, and drug HL, which were all predicted values. The recommended screening and categorization thresholds as used for TCMSP were as follows: OB: ≥30%; DL ≥ 0.18; BBB: <−0.3 is nonpenetrating (BBB-), from −0.3 to + 0.3 represents moderate penetrating (BBB±), and >0.3 represents strong penetrating (BBB+); HL : drug half-life ≤ 4 hours: the fast-elimination group, between 4 and 8 hours are the midelimination group, and ≥8 hours are the slow-elimination group.

Screening of medicine components was based on conventional parameters, and the threshold values were OB ≥ 40% and DL ≥ 0.2.

When there was no small molecule information of herbal chemistry in the TCMSP database, the information in the TCMID database was applied.

### 2.3. Prediction and Screening of Component Target Proteins

The TCMSP and BATMAN (http://bionet.ncpsb.org/batman-tcm/) [21] databases were used to predict the target proteins for our small molecule candidates. BATMAN uses similarity-based methods to predict the potential targets of traditional Chinese medicine components. For our study, we obtained targets from the DrugBank, KEGG, and TTD databases, and BATMAN ranked these target proteins according to interactions between potential targets and similarity of known targets, based on likelihood scores from high to low. The top 50 target proteins with scores >20 were selected.

### 2.4. Cross-Validation of Target Proteins

The Mount Desert Island Biological Laboratory publishes its Comparative Toxicogenomics Database (CTD) (http://ctdbase.org/, updated 2018) [22] to promote understanding of how environmental risks affect human health. In brief, it provides information on chemical-gene/protein interactions and chemical-disease and gene-disease relationships, to help those investigating mechanisms of diseases due to environmental factors.

In order to search the genes related to sexual dysfunction in the CTD database, the “sexual dysfunction” was used as key words, and ranking was conducted based on the inference score [23, 24]. Target proteins with scores >40, which were also target proteins predicted in the previous step, were identified in order to narrow the range of target proteins. Inference scores were calculated using the logarithmic transformation products of two common neighborhood statistics and then used to evaluate the functional correlation between proteins in protein-protein interaction (PPI) networks.

### 2.5. Gene Ontology (GO) and Kyoto Encyclopedia of Genes and Genomes (KEGG) Pathway Analyses

GO functional analysis was performed using the Cytoscape plugins ClueGO + CluePedia [25]. The relevant biological process was selected, and the threshold value was set at P.adjust ≤0.01. Due to the large network, global option GO levels 1–4 were selected, and a functional network was constructed using Cytoscape. The GO tree, being a directed acyclic graph, is a complex structure. To accommodate this, the ClueGO plug-in algorithm enables division of GO items into several levels. When using data without a hierarchy (KEGG, BioCarta), its level is specified as −1. At the first GO levels (1–3), GO data is very common, including many related genes, providing general biological information. GO levels 9–14 operate with specific terms, which have few related genes but a large amount of information, with a higher research value. The kappa coefficient is used for consistency testing or the measurement of classification accuracy; it is calculated based on a confusion matrix [26]. In the ClueGO plugin, the kappa coefficient shows the relationship between GO terms based on overlapping genes. The higher the kappa coefficient, the stronger the term association strength.

KEGG pathway enrichment analysis for target proteins was conducted based on the R package clusterProfiler, with a threshold value of *P* ≤ 0.01.

### 2.6. Protein-Protein Interaction (PPI) Analysis

PPI analysis was performed using the STRING database (version: 10.0, http://www.string-db.org/) [27]. A required confidence level (combined score) > 0.9 was selected as the threshold value, and the relevant tsv format files were downloaded. Cytoscape software was applied to construct the network.

### 2.7. Construction of Pharmacological Network

Based on the herbal medicine-component-target proteins-pathway data generated by previous steps, a pharmacological network was constructed using Cytoscape. The network showed the pathway regulation mechanisms of related target proteins found for important components in the decoction.

## 3. Results

### 3.1. Composition and Component Screening

The number of chemical constituents identified for each herbal medicine ingredient was as follows: *Rehmannia*, 76; yam, 71; *Cornus officinalis*, 226; Chinese wolfberry, 118; dodder, 29; deer horn gum, 2; *Eucommia*, 147; *Cinnamomum cassia*, 100; *Angelica*, 125; *Aconite*, 5; *Astragalus*, 87; licorice, 17; ginseng, 190; orange peel, 109; *Morinda*, 174; Atractylodes, 55; and Xianling spleen, 4. After screening, herbal components of significance were identified (Table 1).

### 3.2. Prediction and Screening of Component Target Proteins and Cross-Validation

A total of 15 herbal medicine ingredients and 110 important components with target records were obtained after completion of predictions using the BATMAN online tool. The predicted targets of the first 50 for each component and the list of targets with scores >20 were obtained. In total, we obtained 13 herbal medicine ingredients and 64 important components with 1747 targets (containing repetition, see Supplementary [Supplementary-material supplementary-material-1]).

**Figure 1:**
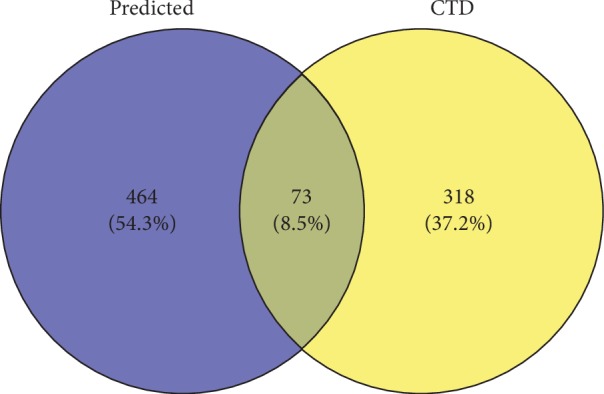
Venn diagrams for cross-validation targets.

These targets were compared with our results for genes relevant to sexual dysfunction with an inference score >40 in the CTD database. We obtained 73 intersection-related targets (Figure 1; Supplementary [Supplementary-material supplementary-material-1]).

### 3.3. GO and KEGG Pathway Analyses

**Figure 2:**
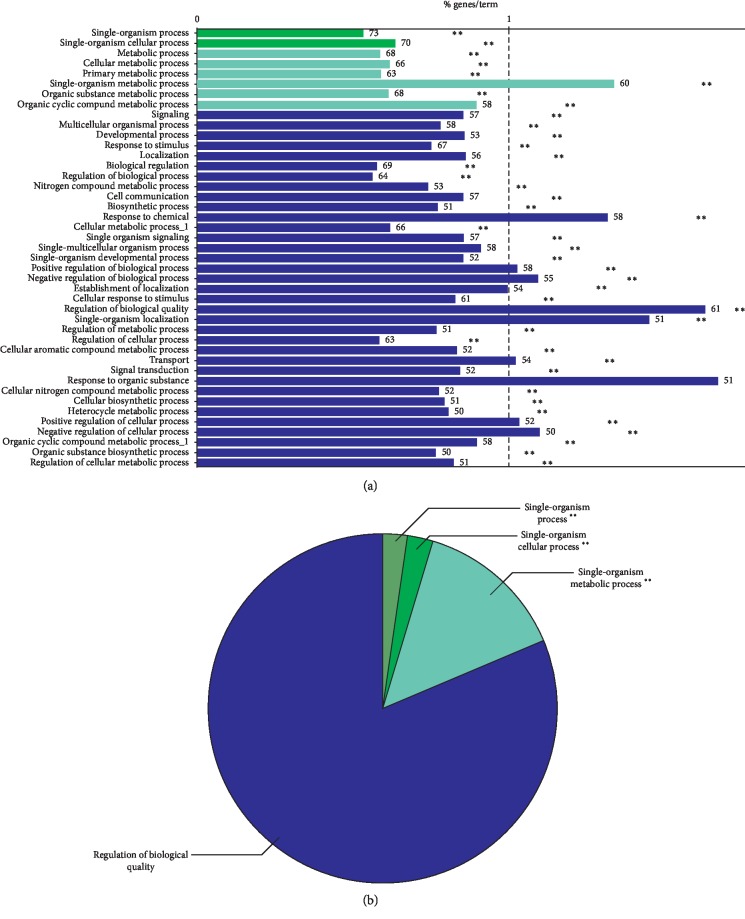
The results of gene ontology (GO) functional analyses: (a) histogram; (b) pie chart. ∗∗ Produced by the analysis software and show no significance.

**Figure 3:**
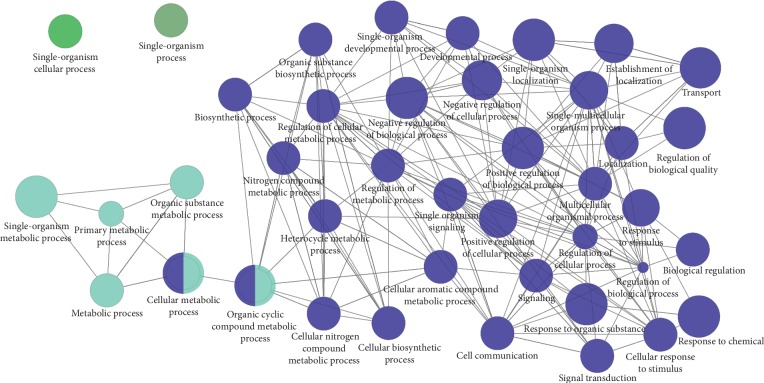
Gene ontology (GO) functional network for 73 target proteins. Nodes: GO terms; bigger nodes indicated smaller *P* values; the two-point line represents the correlation between functions, and the larger the kappa coefficient, the thicker the line.

GO functional analysis showed that a total of 43 GO function items (2 repeats) were enriched (Figure 2(a)), and that the GO function was divided into 4 categories based on the kappa coefficient (Figure 2(b)). Moreover, the GO functional network indicated that the 73 target proteins were enriched in 43 biological processes and classified as either single-organism, single-organism cellular, or single-organism metabolic processes, or regulation of biological quality (Figure 3).

**Figure 4:**
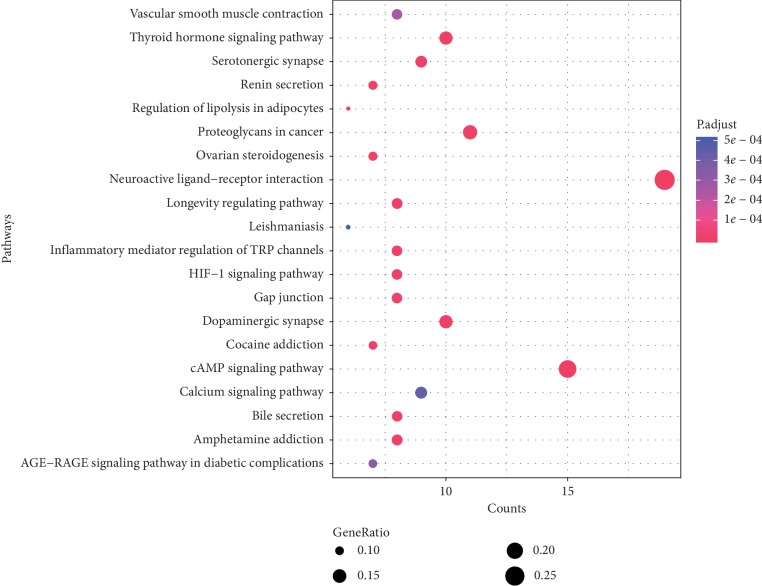
The top 20 pathways. The longitudinal axis: the pathway name; the transverse axis: the number of enriched genes; the size of the dot represents the proportion of the number of enriched genes to the total number of genes, and the larger the proportion is, the larger the dot is; the redder the dot color, the more significant the *P* value.

After pathway analysis using clusterProfiler, a total of 66 pathways were obtained. The top 20 significantly enriched pathways, such as the cAMP signaling pathway, neuroactive ligand-receptor interaction, and proteoglycans in cancer, are illustrated in Figure 4.

### 3.4. PPI Analysis

**Figure 5:**
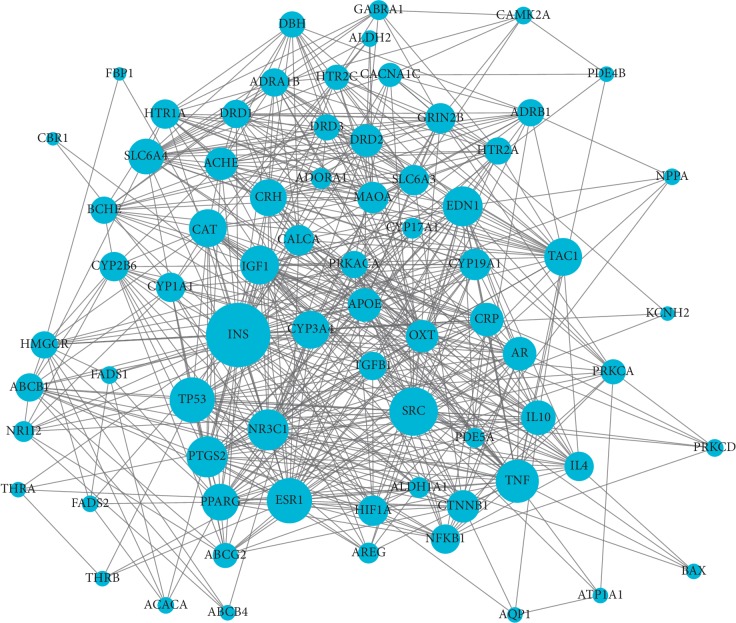
The protein-protein interaction (PPI) network. Dots: target proteins; lines: interactions between proteins; the size of the dot represents the correlation degree with other proteins, and the stronger the correlation degree is, the greater the dot is.

As shown in Figure 5, 73 proteins and 573 relation pairs were found in the PPI network. The top 10 proteins with higher degrees (Supplementary [Supplementary-material supplementary-material-1]) were INS (degree = 50), SRC (degree = 35), TP53 (degree = 32), ESR1 (degree = 32), TNF (degree = 30), PTGS2 (degree = 28), NR3C1 (degree = 28), EDN1 (degree = 27), IGF1 (degree = 26), and CAT (degree = 25); these are potential hub proteins.

### 3.5. Construction of Pharmacological Network

**Figure 6:**
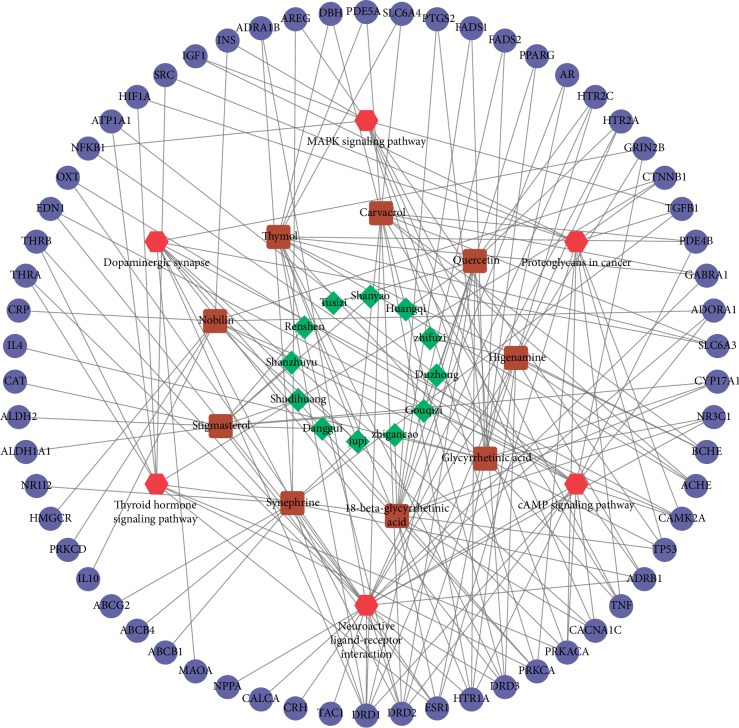
The pharmacological network. Green rhombus: herbal medicine; brown square: components; blue dots: targets; red hexagon: pathways.

Data were refined by retaining only small molecules with a degree ≥10 and pathways with enriched targets ≥10 and by removing nodes without corresponding herbal medicine-component-target proteins-pathways. The processed data were used to construct a pharmacological network. As shown in Figure 6, the network included 89 nodes and 176 relation pairs. Among these nodes, there were 12 herbal medicine nodes (orange peel, licorice, *Eucommia*, *Aconite*, *Astragalus*, Chinese wolfberry, yam, dodder seed, ginseng, *Cornus officinalis*, *Rehmannia*, and *Angelica*), 9 chemical component nodes (18-beta-glycyrrhetinic acid, carvacrol, glycyrrhetinic acid, higenamine, nobilin, quercetin, stigmasterol, synephrine, and thymol), 62 target protein nodes (e.g., NR3C1, ESR1, PTGS2, CAT, TNF, INS, and TP53), and 6 pathway nodes (MAPK signaling pathway, proteoglycans in cancer, dopaminergic synapse, thyroid hormone signaling pathway, cAMP signaling pathway, and neuroactive ligand-receptor interaction).

## 4. Discussion

TCM does not match the chemical drug paradigm of a single component with a single target. It may have a weak effect on a single target, but it can inhibit an entire pathological process through network interaction, playing a unique therapeutic role in maintaining the balance of the body [28]. In this study, following network pharmacology analyses, we identified 12 key herbs (orange peel, licorice, *Eucommia*, *Aconite*, *Astragalus*, Chinese wolfberry, yam, dodder seed, ginseng, *Cornus officinalis*, *Rehmannia*, and *Angelica*) which corresponded to 9 key components (18-beta-glycyrrhetinic acid, carvacrol, glycyrrhetinic acid, higenamine, nobilin, quercetin, stigmasterol, synephrine, and thymol). These 9 key components corresponded in turn to 62 target protein nodes, among which 7 nodes (NR3C1, ESR1, PTGS2, CAT, TNF, INS, and TP53) belonged to hub proteins of the PPI network. Furthermore, the network included 6 pathways: the MAPK signaling pathway, proteoglycans in cancer, dopaminergic synapse activity, the thyroid hormone signaling pathway, the cAMP signaling pathway, and neuroactive ligand-receptor interaction.

The literature was reviewed for relevant activity of the 9 key components. In streptozotocin-induced diabetic rats, quercetin can ameliorate erectile dysfunction [29]. In the process of rat penis erection, quercetin can restore some function of the NO-cGMP pathway [30]. There are no previous studies directly reporting associations between the other 8 key components and sexual dysfunction. However, these 8 components are associated with inflammation. Kao et al. reported that 18-beta-glycyrrhetinic acid and glycyrrhizic acid inhibit inflammation via activation of the glucocorticoid receptor and PI3K/Akt/GSK3 beta-signaling [31]. Fachini-Queiroz et al. suggested that carvacrol and thymol had anti-inflammatory effects [32]. In rat brains with ischemic damage, use of higenamine can reduce inflammation [33]. De Mieri et al. indicated that germacranolide nobilin 1 also had anti-inflammatory effects [34]. In a guinea pig model of ovalbumin-induced asthma, stigmasterol regulated allergic airway inflammation [35]. Berghe et al. reported that synephrine derivatives could be regarded as anti-inflammatory agents [36]. Furthermore, inflammation plays a significant role in erectile dysfunction [37]. Thus, these 9 components are directly relevant components of the Yougui pill and Buzhong Yiqi decoction used to treat sexual dysfunction.

In the current study, NR3C1, ESR1, PTGS2, CAT, TNF, INS, and TP53 were important target protein nodes for our 9 key components. NR3C1 has been reported as a significant downregulator of inflammation [38]. Several studies suggest that CAT plays significant roles in inflammation [39, 40]. Ham et al. indicated that gain-of-function mutations of TP53 could promote inflammation in glioblastoma [41]; low-grade inflammation plays an important role in the pathogenesis of vasculogenic erectile dysfunction [37]. Vignozzi et al. indicated that the damage done to the developing penis by hyperestrogenism involved estrogen receptors (of which ESR1 is one) [42]; estrogen could also mediate erectile dysfunction induced by metabolic syndrome. Schramek and Waldhauser [43] showed that prostaglandin E1 injection was effective for erectile dysfunction; PTGS2 may have a role here. In patients with erectile dysfunction, TNF-α levels in serum are increased; TNF-*α* levels are inversely related to sexual performance [44]. Insulin resistance is an independent predictor of erectile dysfunction and reducing insulin resistance may be useful for preventing erectile dysfunction [45]. In the diabetic rat, insulin treatment may operate to recover erectile function by restoring expression of a sex hormone receptor [46]. Therefore, NR3C1, ESR1, PTGS2, CAT, TNF, INS, and TP53 may be important target proteins for the decoction of Yougui pill combined with Buzhong Yiqi as used to treat sexual dysfunction.

In addition, our data revealed that the 6 pathways (MAPK signaling pathway, proteoglycans in cancer, dopaminergic synapse, thyroid hormone signaling pathway, cAMP signaling pathway, and neuroactive ligand-receptor interaction) may be candidate key pathways involved in the progression of sexual dysfunction. The MAPK signaling pathway plays a role in some human disorders including neurodegenerative diseases and cancers [47]. Sexual dysfunction is a potential complication of some cancer therapy [48]. Steers indicated that signal transduction molecules might be effective methods for enhancing erectile function [49]. Central dopamine plays key roles in the control of sexual function [50]. We infer that the target proteins of our key components may be relevant to sexual dysfunction via pathways associated with cancer (MAPK signaling pathway and proteoglycans in cancer) and signal transduction (dopaminergic synapse, thyroid hormone signaling pathway, cAMP signaling pathway, and neuroactive ligand-receptor interaction).

In conclusion, NR3C1, ESR1, PTGS2, CAT, TNF, INS, and TP53 may be important target proteins for the key components of the decoction containing Yougui pill and Buzhong Yiqi. Furthermore, the nine key chemical components identified in this study targeted proteins including NR3C1, ESR1, PTGS2, CAT, TNF, INS, and TP53, affecting the MAPK signaling pathway, proteoglycans in cancer, dopaminergic synapse activity, the thyroid hormone signaling pathway, the cAMP signaling pathway, and neuroactive ligand-receptor interaction. These pathways are potentially highly relevant to sexual dysfunction. However, these relationships will need to be substantiated by further research.

## Figures and Tables

**Table 1:** Significant herbal components after screening.

Ingredients	Before (number)	After (number)	Source
*Rehmanniae radix* preparata	76	1	TCMSP
*Rhizoma dioscoreae*	71	9	TCMSP
*Cornus officinalis*	226	8	TCMSP
Lycii fructus	188	21	TCMSP
*Semen cuscutae*	29	7	TCMSP
Pulvis cornu cervi	2	2	TCMID
*Eucommia cortex*	147	20	TCMSP
*Cinnamomum cassia*	100	0	TCMSP
*Angelica sinensis* radix	125	1	TCMSP
Radix Aconiti lateralis praeparata	5	5	TCMID
*Hedysarum multijugum* Maxim	87	15	TCMSP
Radix *Glycyrrhizae preparata*	17	17	TCMID
*Panax ginseng*	190	13	TCMSP
*Citrus reticulata*	109	109	TCMID
*Morinda officinalis* radix	174	12	TCMSP
*Atractylodes macrocephala* Koidz	55	4	TCMSP
*Herba epimedii*	4	4	TCMID
Total	1605	248	

## Data Availability

All the data supporting the results reported in the article can be found within the manuscript.

## Additional Points

*Highlights.* (1) NR3C1, ESR1, PTGS2, CAT, TNF, INS, and TP53 may be important target proteins. (2) Nine chemical components were obtained from Yougui pill and Buzhong Yiqi decoction. (3) Six pathways were critical for mechanisms of Yougui pill and Buzhong Yiqi decoction.

## Supplementary Materials

Supplementary Materials Supplementary Table 1: the 73 intersection-related targets. Supplementary Table 2: the top 10 proteins with higher degrees. Supplementary file 1: 13 herbal medicine and 64 important components with 1747 targets.
