# Supplementary material for: Network Pharmacology of Yougui Pill Combined with Buzhong Yiqi Decoction for the Treatment of Sexual Dysfunction
Source: Evid Based Complement Alternat Med. 2019 Nov 11;2019:1243743. doi: 10.1155/2019/1243743 (PMC6877955; doi:10.1155/2019/1243743)
Supplement: Supplementary Materials — Supplementary Table 1: the 73 intersection-related targets. Supplementary Table 2: the top 10 proteins with higher degrees. Supplementary file 1: 13 herbal medicine and 64 important components with 1747 targets. [file 1243743.f1.zip › 1243743.f1/Supplymentary table_ECAM_2904680.docx]

**Supplymentary table 1**: The 73 intersection related targets.

| **Numbers** | **Intersection related targets** |
| --- | --- |
| 73 | HTR2A  HTR2C  DRD2  HTR1A  DRD3  ACHE  BCHE  CRP  AR  NR3C1  CYP17A1  ESR1  PPARG  FADS2  FADS1  PTGS2  IL4  CACNA1C  TAC1  HIF1A  CRH  AQP1  SLC6A3  SLC6A4  CAT  ALDH2  FBP1  IGF1  PDE5A  ADORA1  DRD1  ALDH1A1  TNF  GABRA1  PDE4B  DBH  THRA  THRB  CBR1  CYP1A1  GRIN2B  TGFB1  SRC  AREG  MAOA  NFKB1  CYP3A4  BAX  ATP1A1  ADRB1  NR1I2  KCNH2  ADRA1B  CYP2B6  ACACA  INS  EDN1  OXT  APOE  PRKACA  HMGCR  PRKCA  PRKCD  TP53  CAMK2A  IL10  CTNNB1  CYP19A1  ABCG2  ABCB4  ABCB1  NPPA  CALCA |

**Supplymentary table 2:** The top 10 proteins with higher degrees

| **Gene** | **Degree** | **Betweenness** | **Closeness** |
| --- | --- | --- | --- |
| INS | 50.0 | 889.9373 | 0.7659575 |
| SRC | 35.0 | 402.75116 | 0.6545454 |
| TP53 | 32.0 | 184.98862 | 0.62608695 |
| ESR1 | 32.0 | 172.3097 | 0.6315789 |
| TNF | 30.0 | 190.01242 | 0.62068963 |
| PTGS2 | 28.0 | 62.231308 | 0.6101695 |
| NR3C1 | 28.0 | 153.30898 | 0.61538464 |
| EDN1 | 27.0 | 119.53957 | 0.6 |
| IGF1 | 26.0 | 56.799942 | 0.59016395 |
| CAT | 25.0 | 230.19742 | 0.59504133 |
